# Economic and health impacts of *Helicobacter pylori* eradication strategy for the treatment of peptic ulcer disease: A cost‐effectiveness analysis

**DOI:** 10.1111/hel.12886

**Published:** 2022-03-27

**Authors:** Akiko Kowada, Masahiro Asaka

**Affiliations:** ^1^ Advanced Research Promotion Center Health Sciences University of Hokkaido Ishikari‐gun Japan; ^2^ Department of Occupational Health Kitasato University Graduate School of Medical Sciences Kanagawa Japan

**Keywords:** economics, eradication, *Helicobacter pylori*, peptic ulcer disease, prevention, proton pump inhibitor

## Abstract

**Background:**

Most peptic ulcer cases are associated with *Helicobacter pylori* (*H*. *pylori*) infection or the use of nonsteroidal anti‐inflammatory drugs (NSAIDs). *H*. *pylori* eradication therapy is recommended for the treatment of *H*. *pylori*‐positive peptic ulcers. We aimed to assess and validate the cumulative economic and health effects of *H*. *pylori* eradication strategy for the treatment of peptic ulcers compared with PPI therapy strategy.

**Materials and Methods:**

We developed a cohort state‐transition model for *H*. *pylori* eradication strategy and PPI therapy strategy over a lifetime horizon from a healthcare payer perspective. We targeted two hypothetical cohorts of *H*. *pylori*‐positive patients with gastric and duodenal ulcers aged 20, 30, 40, 50, 60, 70, and 80. The main outcomes were costs, quality‐adjusted life‐years (QALYs), life expectancy life‐years (LYs), incremental cost‐effectiveness ratios, ulcer recurrence cases, and ulcer‐associated deaths. One‐way and probabilistic sensitivity analyses were conducted to assess the impact of uncertainty.

**Results:**

In the base‐case analysis, *H*. *pylori* eradication strategy was less costly with greater benefits than PPI therapy strategy in all age groups. Cost‐effectiveness was not sensitive to any variables in all age groups. Sensitivity analyses showed strong robustness of the results. From 2000 to 2020, *H*. *pylori* eradication strategy saved US$14.07 billion over a lifetime, increased 8.65 million QALYs and 1.23 million LYs over a lifetime, and prevented 551,298 ulcer recurrence cases and 59,465 ulcer‐associated deaths, compared with PPI therapy strategy.

**Conclusions:**

*H*. *pylori* eradication strategy not only has contributed significantly to preventing ulcer recurrence and reducing ulcer‐associated deaths but also has resulted in great cost savings. All over the world, *H*. *pylori* eradication strategy is likely to have yielded a comparable magnitude of economic and health benefits, depending on the epidemiology of *H*. *pylori*‐related peptic ulcers and the healthcare environment in each country.

## 1 INTRODUCTION

The decreasing trend of peptic ulcer disease occurs over the past two decades in the world.^1^ This decrease is considered to be mainly due to the decline in the prevalence of *Helicobacter pylori* (*H*. *pylori*) infection, widespread use of proton pump inhibitors (PPIs), and appropriate use of nonsteroidal anti‐inflammatory drugs (NSAIDs) and aspirin.^2^, ^3^, ^4^ Peptic ulcer‐associated mortality has also decreased significantly and shown birth‐cohort phenomenon.^1^, ^5^

A majority of peptic ulcer cases are associated with *H*. *pylori* infection or the use of nonsteroidal anti‐inflammatory drugs. PPIs substantially changed the approach to peptic ulcer disease management and improved the rate of peptic ulcer healing. *H*. *pylori* eradication therapy is recommended for the treatment of *H*. *pylori*‐positive peptic ulcers.

**FIGURE 1:**
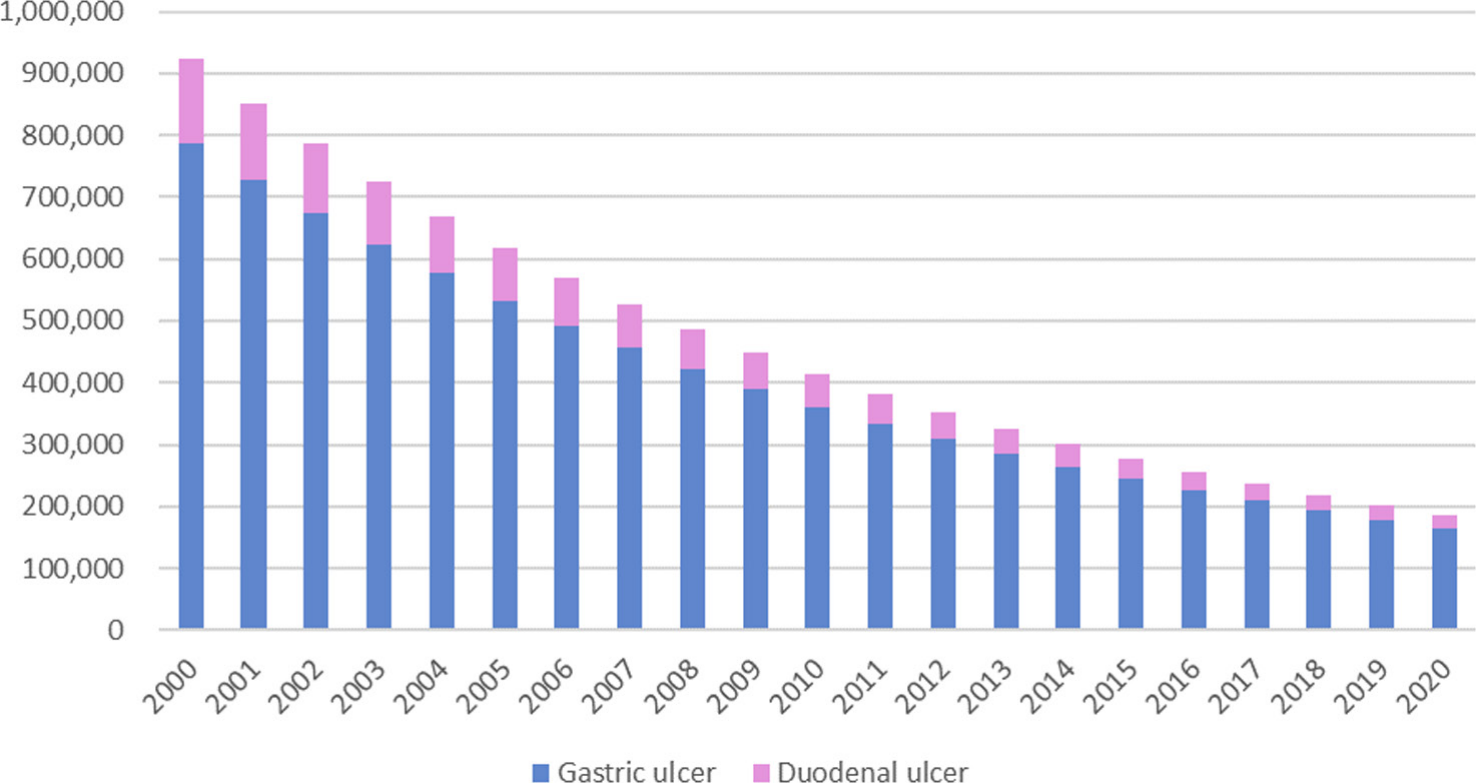
Number of patients with gastric and duodenal ulcer in Japan based on patient surveys from 2000 to 2020

*H*. *pylori* infection accounts for more than 90% of the causes of peptic ulcers in Japan.^6^, ^7^ In 2000, the Ministry of Health, Labour, and Welfare (MHLW) approved the National Health Insurance coverage of *H*. *pylori* eradication therapy for patients with peptic ulcer disease. The evidence‐based clinical practice guidelines for peptic ulcer disease by the Japanese Society of Gastroenterology recommend *H*. *pylori* eradication therapy for the treatment of *H*. *pylori*‐positive peptic ulcers.^8^ Since 2000, *H*. *pylori* eradication tharapy has replaced PPI therapy for the treatment of peptic ulcer disease in Japan. Based on patient surveys, there were 9.4 million peptic ulcer patients between the ages of 20 and 89 from 2000 to 2020, and it is estimated that 8.46 million peptic ulcer patients eradicated *H*. *pylori*. The number of patients with peptic ulcers has decreased by one‐fifth, from 923,983 in 2000 to 185,891 in 2020 (Figure 1). It is now very important to assess and validate the economic and health benefits of *H*. *pylori* eradication strategy in the treatment of peptic ulcers. Cost‐effectiveness regarding *H*. *pylori* eradication strategy warrants evaluation as a healthcare policy that has been implemented for the management of peptic ulcers.

In this study, we aimed to assess and validate the cumulative economic and health effects of *H*. *pylori* eradication strategy for the treatment of peptic ulcer disease.

## 2 MATERIALS AND METHODS

### 2.1 Study design and model structure

**FIGURE 2:**
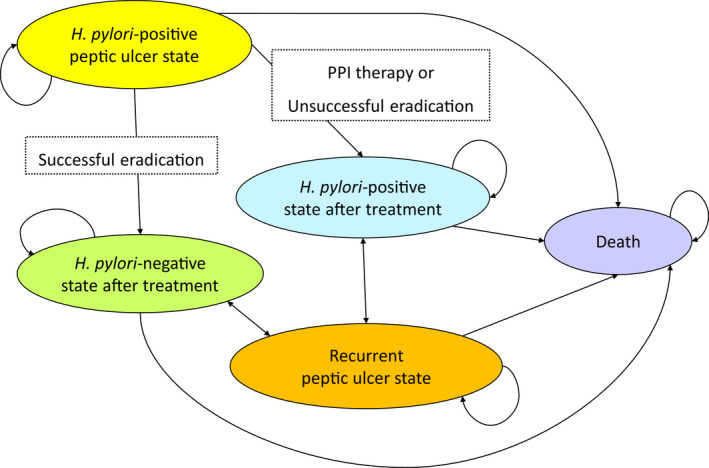
Simplified schematic depiction of a state‐transition diagram. We show health states in the model as ovals. In a yearly model cycle, transition paths can occur between the health states and other health states, as represented by the arrows. *H*. *pylori*, *Helicobacter pylori*

The analysis was conducted from a healthcare payer perspective and a lifetime horizon. We constructed a cohort state‐transition model for two intervention strategies: *H*. *pylori* eradication strategy and PPI therapy strategy. A simplified schematic depiction of a state‐transition diagram is shown in Figure 2, including five health states and possible transition paths. Decision branches led directly to one Markov node per intervention strategy, and the first event was modeled within a Markov cycle tree. A cycle length of one year was chosen. The half‐cycle correction was applied. Incremental cost‐effectiveness ratios (ICERs) were calculated and compared with the willingness‐to‐pay (WTP) levels of US$50,000 per quality‐adjusted life‐year (QALY) gained and US$100,000 per QALY gained.^9^

The main outcome measures were costs, QALYs, life expectancy life‐years (LYs), ICERs, ulcer recurrence cases, and ulcer‐associated deaths.

As this was a modeling study with all inputs and parameters derived from the published literature and Japanese statistics, ethics approval was not required. We constructed the model using TreeAge Pro 2022 (TreeAge Software Inc., Williamstown, Massachusetts).

#### 2.1.1 *H. pylori* eradication strategy

*H*. *pylori*‐positive patient with gastric ulcer (GU) or duodenal ulcer (DU) receives first‐line *H*. *pylori* eradication therapy (vonoprazan 40 mg/day, clarithromycin 400 mg/day, and amoxicillin 1500 mg/day for 7 days).^8^ At the start of *H*. *pylori* eradication therapy, we added the cost of one *H*. *pylori* test, one endoscopy, and two urea breath tests. The patient who fails first‐line *H*. *pylori* eradication therapy receives second‐line *H*. *pylori* eradication therapy (vonoprazan 40 mg/day, metronidazole 500 mg/day, and amoxicillin 1500 mg/day for 7 days).^8^ After successful *H*. *pylori* eradication, *H*. *pylori* positive changes to *H*. *pylori* negative. When the patient fails both treatments and *H*. *pylori* is not eradicated, *H*. *pylori* positive remains and the patient receives PPI therapy. PPI therapy consists of 30 mg/day of lansoprazole for 8 weeks in patients with GU and 6 weeks in patients with DU, based on the third edition of evidence‐based clinical practice guidelines for peptic ulcer disease by the Japanese Society of Gastroenterology.^8^ When ulcer recurrence occurs after *H*. *pylori* eradication, the patient repeats PPI therapy and endoscopy.

We considered the eradication success rate, the compliance rate of first‐line and second‐line *H*. *pylori* eradication therapies, and the recurrence rate of peptic ulcers in the model.

#### 2.1.2 PPI therapy strategy

*H*. *pylori*‐positive patient with GU and DU receives endoscopy and PPI therapy. PPI therapy consists of 30 mg/day of lansoprazole for 8 weeks in patients with GU and 6 weeks in patients with DU. When ulcer recurrence occurs, the patient repeats PPI therapy and endoscopy.

### 2.2 Target population

We targeted two hypothetical cohorts of *H*. *pylori*‐positive patients with GU and DU aged 20, 30, 40, 50, 60, 70, and 80. We obtained the total number of patients with peptic ulcer disease every three years from patient surveys^10^ and estimated the annual number of *H*. *pylori*‐positive patients with GU and DU by age group from 2000 to 2020 (Supplementary Table 1).^10^

### 2.3 Model inputs

#### 2.3.1 Clinical probabilities

Clinical probabilities were collected using MEDLINE from 1980 to February 2021 (Table 1). The eradication success rates of first‐ and second‐line *H*. *pylori* eradication therapies, the compliance rates of first‐ and second‐line *H*. *pylori* eradication therapies, *H*. *pylori* positivity rate in patients of peptic ulcer disease, and the ulcer recurrence rate were obtained from the literature.^6^, ^7^, ^11^, ^12^ The mortality from other causes and the ulcer‐associated death rate were obtained from vital statistics.^13^

**TABLE 1:** Model inputs for selected variables

Variable	Baseline value	Sensitivity analysis range	References
Probabilities			
Eradication success rate of first‐line *H*. *pylori* eradication therapy	0.798	0.6–1.0	11
Eradication success rate of second‐line *H*. *pylori* eradication therapy	0.837	0.6–1.0	11
Compliance rate of first‐line *H*. *pylori* eradication therapy	0.848	0.6–1.0	11
Compliance rate of second‐line *H*. *pylori* eradication therapy	0.678	0.6–1.0	11
Recurrence rate of peptic ulcer in *H. pylori* eradication strategy	0.129	0.077–0.211	12
Recurrence rate of peptic ulcer in PPI therapy strategy	0.247	0.15–0.404	12
Ulcer‐associated death rate	0.0078	0.0042–0.0127	13
Costs, US$ (US$1=¥ 102.835)			
*H*. *pylori* test	7.8	5.9–9.8	14
Urea breath test	6.8	5.1–8.5	
First‐line *H*. *pylori* eradication therapy	41.9	31.4–52.4	
Second‐line *H*. *pylori* eradication therapy	38.1	28.6–47.6	
Endoscopy	110.9	83.2–138.6	
PPI therapy for gastric ulcer	49.4	37.1–61.8	
PPI therapy for duodenal ulcer	37.1	27.8–46.4	
Utilities			
*H*. *pylori*‐positive peptic ulcer state	0.89	0.87–0.91	18
*H*. *pylori*‐negative state after treatment	0.99	0.95–1	
*H*. *pylori*‐positive state after treatment	0.91	0.89–0.93	
Recurrent peptic ulcer state	0.89	0.87–0.91	
Death	0	N/A	

Note

Abbreviations: *H*. *pylori*, *Helicobacter pylori*; N/A, not applicable; PPI, proton pump inhibitor.

#### 2.3.2 Costs

Costs were calculated based on the costs listed in the Japanese medical fee schedule^14^ and adjusted to 2020 Japanese yen, using the medical care component of the Japanese consumer price index, and converted to 2020 US dollars, using the Organisation for Economic Co‐operation and Development (OECD) purchasing power parity rate (US$1=¥102.835) (Table 1).^14^, ^15^ All direct costs were based on healthcare payer perspectives. All costs were discounted by 3%.^16^, ^17^

#### 2.3.3 Health utilities

Health status was included to represent possible five clinical states: (i) *H*. *pylori*‐positive peptic ulcer state, (ii) *H*. *pylori*‐positive state after treatment, (iii) *H*. *pylori*‐negative state after treatment, (iv) recurrent peptic ulcer state, and (v) death (Figure 2). Health state utilities were obtained from the literature^18^ and were calculated using utility weights with values ranging from 1 (healthy) to 0 (death) (Table 1). The annual discounting of the utilities was set at a rate of 3%.^16^, ^17^

### 2.4 Sensitivity analysis

We conducted a one‐way sensitivity analysis to determine which strategy was more cost‐effective when a single variable was tested over the widest possible range, holding all other variables constant. The variables in the one‐way sensitivity analysis are shown in Table 1. The variables such as the cost of *H*. *pylori* eradication therapy, the cost of PPI therapy, the eradication success rate, the compliance rate of *H*. *pylori* eradication therapy, and the recurrence rate of peptic ulcer were considered. The ICER tornado diagram was created to show the changing incremental value between PPI therapy strategy versus *H*. *pylori* eradication strategy for each key parameter. To assess the impact of model uncertainty on the base‐case estimates, we also performed the probabilistic sensitivity analysis using a second‐order Monte Carlo simulation over 10,000 trials. The uncertainty had a beta distribution for probability and accuracy, and a gamma distribution for cost.

### 2.5 Scenario analysis

We performed a scenario analysis by varying the *H*. *pylori* positivity rate in patients with peptic ulcers in the range of 0.7 to 1.0. We calculated the cumulative lifetime cost savings, cumulative lifetime QALY gains, cumulative lifetime LY gains, cumulative ulcer recurrence cases prevented, and cumulative ulcer‐associated deaths prevented between 2000 and 2020.

### 2.6 Markov cohort analysis

In the Markov cohort analysis, we determined the cumulative probability of peptic ulcer recurrence prevented and the cumulative probability of ulcer‐associated death prevented between 2000 and 2020 comparing *H*. *pylori* eradication strategy and PPI therapy strategy in each age group.

### 2.7 Cumulative economic and health outcomes

The cumulative lifetime cost savings and cumulative lifetime effectiveness of *H*. *pylori* eradication strategy versus PPI therapy strategy were calculated by multiplying the age‐specific incremental cost and age‐specific incremental effectiveness by the total age‐specific number of *H*. *pylori*‐positive ulcer patients between 2000 and 2020 and then summing them. The cumulative ulcer recurrence case prevented by *H*. *pylori* eradication strategy between 2000 and 2020 was calculated by multiplying the age‐specific cumulative probability of ulcer recurrence prevented for each year by the annual age‐specific number of *H*. *pylori*‐positive ulcer patients and then summing them. The cumulative ulcer‐associated death prevented by *H*. *pylori* eradication strategy between 2000 and 2020 was calculated by multiplying the age‐specific cumulative probability of ulcer‐associated death prevented for each year by the annual age‐specific number of *H*. *pylori*‐positive ulcer patients and then summing them.

## 3 RESULTS

### 3.1 Base‐case analysis

*H*. *pylori* eradication strategy was less costly and yielded greater benefits than PPI therapy strategy in all age groups (Table 2). PPI therapy strategy was dominated by *H*. *pylori* eradication strategy in all age groups (Table 2). Per capita cost savings, QALY gain, and LY gains of *H*. *pylori* eradication strategy were higher in younger than in older age groups (Table 2).

**TABLE 2:** Base‐case analysis

Age group (y)	Strategy	GU cost (US$)	GU incremental cost (US$)	DU cost (US$)	DU incremental cost (US$)	Effectiveness (QALYs)	Incremental QALYs	ICER (US$/QALY gained)	Effectiveness (LYs)	Incremental LYs	ICER (US$/LY gained)
20	*H*. *pylori* eradication	1,554	–	1,449	–	25.924	–	–	27.011	–	–
	PPI therapy	4,347	2,792	4,013	2,564	24.081	−1.843	Dominated	26.615	−0.396	Dominated
30	*H*. *pylori* eradication	1,465	–	1,366	–	24.234	–	–	25.253	–	–
	PPI therapy	4,075	2,610	3,762	2,396	22.547	−1.686	Dominated	24.921	−0.332	Dominated
40	*H*. *pylori* eradication	1,345	–	1,256	–	21.995	–	–	22.925	–	–
	PPI therapy	3,713	2,368	3,428	2,172	20.503	−1.492	Dominated	22.662	−0.263	Dominated
50	*H*. *pylori* eradication	1,193	–	1,115	–	19.146	–	–	19.960	–	–
	PPI therapy	3,249	2,057	3,000	1,885	17.885	−1.261	Dominated	19.769	−0.191	Dominated
60	*H*. *pylori* eradication	1,007	–	944	–	15.700	–	–	16.375	–	–
	PPI therapy	2,685	1,678	2,479	1,535	14.701	−0.999	Dominated	16.252	−0.123	Dominated
70	*H*. *pylori* eradication	788	–	742	–	11.637	–	–	12.147	–	–
	PPI therapy	2,017	1,228	1,862	1,120	10.927	−0.710	Dominated	12.082	−0.066	Dominated
80	*H*. *pylori* eradication	560	–	531	–	7.419	–	–	7.758	–	–
	PPI therapy	1,320	759	1,218	687	6.990	−0.429	Dominated	7.732	−0.026	Dominated

Note

Abbreviations: DU, duodenal ulcer; GU, gastric ulcer; *H*. *pylori*, *Helicobacter pylori*; ICER, incremental cost‐effectiveness ratio; LY, life expectancy life‐year; PPI, proton pump inhibitor; QALY, quality‐adjusted life‐year; Dominated, less effective, and more costly than others.

### 3.2 Sensitivity analysis

**FIGURE 3:**
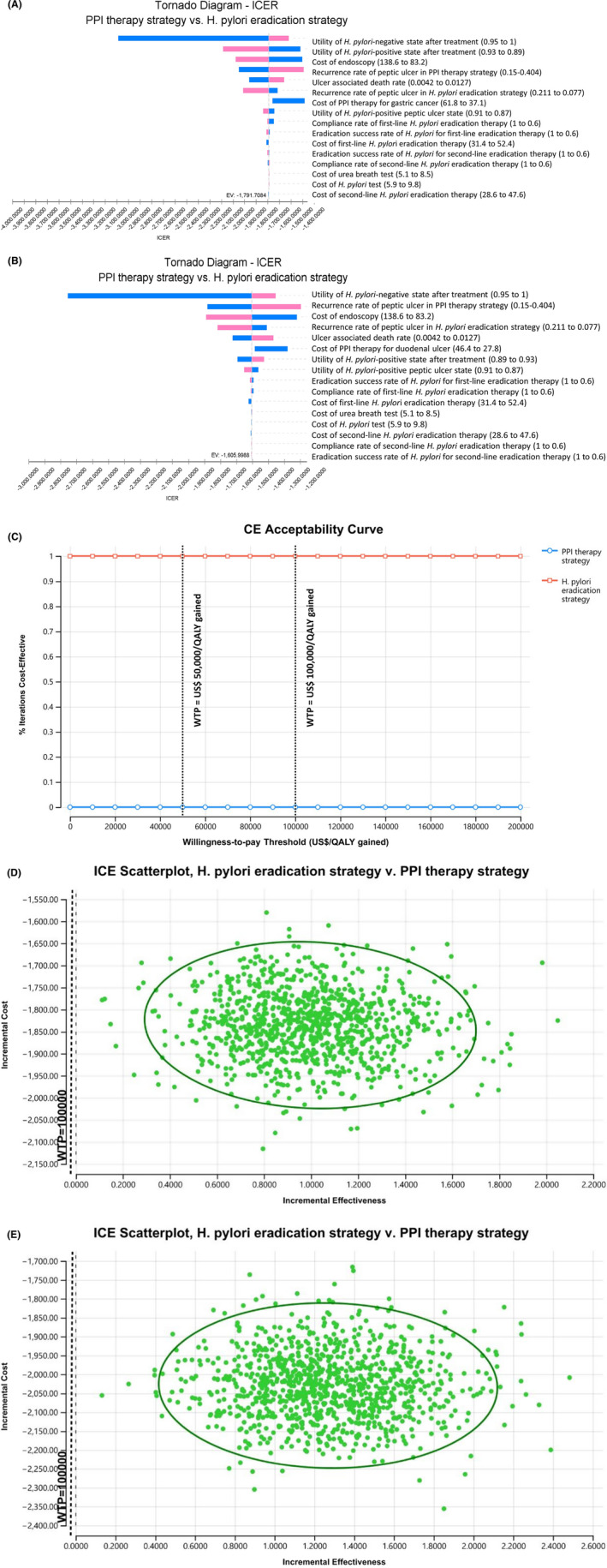
Sensitivity analyses. (A) Incremental cost‐effectiveness ratio (ICER) tornado diagram for PPI therapy strategy versus *H*. *pylori* eradication strategy in 60‐year‐old patients with gastric ulcer. (B) ICER tornado diagram for PPI therapy strategy versus *H*. *pylori* eradication strategy in 50‐year‐old patients with duodenal ulcer. Both ICER tornado diagrams showed that the cost‐effectiveness was not sensitive to any variables. (C) Cost‐effectiveness acceptability curve. The probabilistic sensitivity analysis analyzed 10,000 simulations of the model in which input parameters were randomly varied across prespecified statistical distributions. The x‐axis represents the willingness‐to‐pay (WTP) threshold. The acceptability curve showed that *H*. *pylori* eradication strategy was cost‐effective 100% of the time at two willingness‐to‐pay thresholds of US$50,000 per QALY gained and US$100,000 per QALY gained. (D) Incremental cost‐effectiveness (ICE) scatterplot with a 95% confidence ellipse in 60‐year‐old patients with gastric ulcer. (E) ICE scatterplot with a 95% confidence ellipse in 50‐year‐old patients with duodenal ulcer. Each dot represents a single simulation for a total of 10,000 simulations. Both ICE scatterplots showed that *H*. *pylori* eradication strategy was dominant to PPI therapy strategy in 10,000 trials. EV, expected value; *H*. *pylori*, *Helicobacter pylori*; ICE, incremental cost‐effectiveness; ICER, incremental cost‐effectiveness ratio; PPI, proton pump inhibitor; QALY, quality‐adjusted life‐year; WTP, willingness to pay

One sensitivity analysis showed that cost‐effectiveness was not sensitive to any variables such as cost of *H*. *pylori* eradication therapy, cost of PPI therapy, eradication success rates, compliance rates of *H*. *pylori* eradication therapy, and recurrence rates of peptic ulcer in all age groups. The ICER tornado diagrams of PPI therapy strategy versus *H*. *pylori* eradication strategy also showed that cost‐effectiveness was not sensitive to any variable in all age groups of GU and DU patients, and are shown in Figure 3A,B.

In the probabilistic sensitivity analysis using a second‐order Monte Carlo simulation for 10,000 trials, the acceptability curves showed that *H*. *pylori* eradication strategy was cost‐effective 100% of the time at two willingness‐to‐pay thresholds of US$50,000 per QALY gained and US$100,000 per QALY gained in all age groups of GU and DU patients (Figure 3C).

The incremental cost‐effectiveness scatterplots showed that *H*. *pylori* eradication strategy was dominant to PPI therapy strategy in 10,000 trials in all age groups of GU and DU patients (Figure 3D, E).

### 3.3 Scenario analysis

The scenario analysis showed that varying the *H*. *pylori* positivity rates among peptic ulcer patients between 70% and 100% resulted in cumulative lifetime cost savings of US$10.94 billion to US$15.63 billion, cumulative ulcer recurrence cases prevented of 428,788 to 612,554, and cumulative ulcer‐associated deaths prevented of 46,250 to 66,072 (Table 3).

**TABLE 3:** Scenario analysis on *H*. *pylori* positivity rate in patients with peptic ulcer disease

Age group (y)	Cumulative lifetime cost savings in GU patients (US$)	Cumulative lifetime QALY gains in GU patients (QALYs)	Cumulative lifetime LY gains in GU patients (LYs)	Cumulative lifetime cost savings in DU patients (US$)	Cumulative lifetime QALY gains in DU patients (QALYs)	Cumulative lifetime LY gains in DU patients (LYs)	Cumulative ulcer recurrence cases prevented (2000–2020)	Cumulative ulcer‐associated deaths prevented (2000–2020)
*H*. *pylori* positivity rate = 1.0								
20	645,772,915	426,296	91,642	89,408,416	64,275	13,817	22,675	2,172
30	1,191,601,513	769,795	151,737	264,599,913	186,221	36,707	48,031	4,627
40	1,974,329,425	1,244,260	218,957	479,721,998	329,560	57,994	87,845	8,536
50	3,016,735,330	1,849,456	279,999	514,876,002	344,418	52,143	139,250	13,736
60	3,319,136,456	1,975,274	243,646	401,524,908	261,166	32,214	162,394	16,776
70	2,564,724,501	1,481,756	136,853	247,423,885	156,757	14,478	118,587	14,874
80	845,723,194	477,414	28,992	75,872,710	47,332	2,874	33,772	5,351
Total	13,558,023,334	8,224,250	1,151,826	2,073,427,832	1,389,729	210,228	612,554	66,072
*H*. *pylori* positivity rate = 0.9								
20	581,195,624	383,666	82,478	80,467,575	57,847	12,436	20,407	1,955
30	1,072,441,362	692,816	136,563	238,139,921	167,599	33,036	43,228	4,164
40	1,776,896,482	1,119,834	197,062	431,749,799	296,604	52,195	79,060	7,682
50	2,715,061,797	1,664,510	251,999	463,388,402	309,976	46,929	125,325	12,363
60	2,987,222,810	1,777,746	219,281	361,372,417	235,050	28,993	146,155	15,098
70	2,308,252,051	1,333,580	123,168	222,681,497	141,081	13,030	106,728	13,387
80	761,150,875	429,672	26,092	68,285,439	42,599	2,587	30,395	4,816
Total	12,202,221,000	7,401,825	1,036,643	1,866,085,049	1,250,756	189,205	551,298	59,465
*H*. *pylori* positivity rate = 0.8								
20	516,618,332	341,037	73,314	71,526,733	51,420	11,054	18,140	1,738
30	953,281,210	615,836	121,390	211,679,930	148,977	29,365	38,425	3,701
40	1,579,463,540	995,408	175,166	383,777,599	263,648	46,395	70,276	6,829
50	2,413,388,264	1,479,565	223,999	411,900,801	275,534	41,715	111,400	10,989
60	2,655,309,165	1,580,219	194,917	321,219,927	208,933	25,771	129,915	13,420
70	2,051,779,601	1,185,405	109,482	197,939,108	125,405	11,582	94,869	11,899
80	676,578,555	381,931	23,193	60,698,168	37,866	2,299	27,018	4,281
Total	10,846,418,667	6,579,400	921,461	1,658,742,265	1,111,783	168,182	490,043	52,858
*H*. *pylori* positivity rate =0.7								
20	452,041,041	298,407	64,149	62,585,891	44,992	9,672	15,872	1,521
30	834,121,059	538,857	106,216	185,219,939	130,355	25,695	33,622	3,239
40	1,382,030,597	870,982	153,270	335,805,399	230,692	40,596	61,491	5,975
50	2,111,714,731	1,294,619	195,999	360,413,201	241,092	36,500	97,475	9,615
60	2,323,395,519	1,382,691	170,552	281,067,436	182,816	22,550	113,676	11,743
70	1,795,307,151	1,037,229	95,797	173,196,720	109,730	10,134	83,011	10,412
80	592,006,236	334,190	20,294	53,110,897	33,133	2,012	23,641	3,746
Total	9,490,616,334	5,756,975	806,278	1,451,399,482	972,810	147,159	428,788	46,250

Note

Abbreviations: DU, duodenal ulcer; GU, gastric ulcer; *H*. *pylori*, *Helicobacter pylori*; LY, life expectancy life‐year; PPI, proton pump inhibitor; QALY, quality‐adjusted life‐year.

### 3.4 Cumulative economic and health outcomes

Between 2000 and 2020, *H*. *pylori* eradication strategy saved US$14.07 billion over a lifetime, increased 8.65 million QALYs and 1.23 million LYs over a lifetime, and prevented 551,298 ulcer recurrence cases and 59,465 ulcer‐associated deaths, compared with PPI therapy strategy (Table 3). GU patients aged 60 and DU patients aged 50 had the highest lifetime cumulative cost savings, lifetime cumulative QALYs gains, and lifetime cumulative LY gains in all age groups. Between 2000 and 2020, among all age groups, patients with peptic ulcers aged 60 years had the highest number of cumulative ulcer recurrences and cumulative ulcer‐associated deaths prevented by *H*. *pylori* eradication strategy (Table 3).

## 4 DISCUSSION

We previously demonstrated that *H*. *pylori* eradication strategy for gastric cancer prevention is cost‐effective^19^, ^20^, ^21^ and provides significant cumulative lifetime cost savings and great cumulative lifetime health benefits.^21^ This study suggests that *H*. *pylori* eradication strategy provides significant cost savings for *H*. *pylori*‐positive patients with peptic ulcers, and contributes to the reduction in peptic ulcer morbidity and mortality compared with PPI therapy strategy. To the best of our knowledge, this is the first modeling study to assess the cumulative economic and health effects of *H*. *pylori* eradication strategy compared with PPI therapy strategy for the treatment of peptic ulcer disease in the world.

Recently, the use of NSAIDs, aspirin, and anti‐thrombotic drugs is increasing in older people with cardiovascular and cerebrovascular diseases and has become a more important risk factor for peptic ulcers.^1^ Older people who habitually take NSAIDs, aspirin, and anti‐thrombotic drugs often have concurrent *H*. *pylori* infection.^1^ NSAIDs or aspirin‐associated peptic ulcers cause the major complications of peptic ulcers, including bleeding, perforation, and gastric outlet obstruction, which lead to hospitalization. PPI therapy is superior to *H*. *pylori* eradication therapy in preventing recurrent bleeding in patients taking NSAIDs.^22^ It is recommended to introduce *H*. *pylori* eradication therapy in *H*. *pylori*‐positive patients before starting long‐term prophylactic treatment with NSAIDs and aspirin because *H*. *pylori* eradication therapy reduces the incidence of future ulcers and gastric cancer.^8^, ^23^, ^24^ The results of this study support that *H*. *pylori* eradication strategy for elderly patients taking NSAIDs, aspirin, and anti‐thrombotic drugs may result in cost savings by reducing the incidence of peptic ulcers. Idiopathic peptic ulcer without *H*. *pylori* infection and without the use of NSAIDs has high mortality and is gradually increasing, and its control is a major issue for the future.^1^, ^25^, ^26^, ^27^

There are several cost‐effectiveness studies of *H*. *pylori* eradication strategy for the treatment of peptic ulcers. Ikeda et al^28^ demonstrated that *H*. *pylori* eradication triple therapy was less costly and more effective than histamine‐2 receptor antagonist therapy for the treatment of peptic ulcers in Japan. Sonnenberg and Everhart showed that expenditures attributed to peptic ulcers, with significant damage to patients’ health, amounted to US$5.65 billion per year in the United States in 1989.^29^ Eslick et al^30^ found that triple therapy saved AU$10.03 billion including direct and indirect costs, prevented 18,665 deaths, and saved 258,887 life‐years and 33,776 productive life‐years in Australia between 1990 and 2015. They calculated indirect costs associated with excess mortality using a range of techniques and direct costs using the annual number of hospitalizations for peptic ulcer disease obtained from data in the National Hospital Morbidity Database in Australia and the cost of each hospitalization event based on data from the National Hospital Cost Data Collection. In Eslick's study, the method of cost calculation was quite different from our modeling study. In addition, the prevalence of *H*. *pylori* in Australia was 24.6% (17.2–32.1), which was lower than 51.7% (44.7–58.7) in Japan.^31^

This study has several limitations. First, we predicted the annual number of peptic ulcer patients based on a triennial patient survey. Second, *H*. *pylori* positivity rate in patients of peptic ulcer disease was obtained from the literature.^6^, ^7^ Third, the costs did not take into account the hospitalization or complications from peptic ulcers, such as peptic ulcer bleeding and outlet obstruction in the model. This may lead to an underestimation of relative cost‐effectiveness results. Fourth, nonmedical indirect costs, such as lost productivity, work absenteeism, and income loss, were not included in this study. Fifth, we did not consider reinfection and recurrence of *H*. *pylori* infection in the model. The reinfection rate after *H*. *pylori* eradication is very low. *H*. *pylori* infection is mainly transmitted in childhood, and recurrence of *H*. *pylori* infection after successful eradication is rare in adults.^32^ Sixth, the costs for *H*. *pylori* eradication regimens and PPI therapy were based on the costs covered by the National Health Insurance in Japan. To improve the generalizability, we performed a one‐way sensitivity analysis on these costs and showed that cost‐effectiveness was not sensitive to these costs. Seventh, the ulcer recurrence rate was set similarly across different age groups in the model. The older age population may have a higher recurrence rate due to other etiologies, affecting the magnitude of relative cost‐effectiveness. Finally, the study limited its focus to peptic ulcers and did not take into account gastric cancer or functional dyspepsia.

## 5 CONCLUSION

This modeling study suggests that *H*. *pylori* eradication strategy not only has contributed significantly to preventing ulcer recurrence and reducing ulcer‐associated deaths but also has resulted in great cost savings. The findings strongly support that *H*. *pylori* eradication strategy has significant economic and health benefits as a healthcare policy for the treatment of peptic ulcers in high‐prevalence countries worldwide.

## CONFLICT OF INTEREST

The authors have no conflicts of interest to declare.

## AUTHOR CONTRIBUTION

AK had full access to all the data in the study and takes responsibility for the integrity of the data and the accuracy of the data analysis. AK and MA approved the final version of the manuscript. AK and MA conceptualized and designed the study, and critically revised the manuscript for important intellectual content. AK acquired the data, analyzed the data, interpreted the data, drafted the manuscript, and provided administrative, technical, or material support. MA supervised the study.

## Supporting information

Supplementary Material
