# Supplementary material for: Economic and health impacts of Helicobacter pylori eradication strategy for the treatment of peptic ulcer disease: A cost‐effectiveness analysis
Source: Helicobacter. 2022 Mar 27;27(3):e12886. doi: 10.1111/hel.12886 (PMC9286595; doi:10.1111/hel.12886)
Supplement: Supplementary file 1 — Supplementary Material [file HEL-27-0-s001.pdf]

**SUPPLEMENTARY MATERIAL**

**Supplementary Table 1.** Annual number of patients with peptic ulcer disease by age group, Japan 2000-2020

| Number of patients with gastric ulcer (n) | | | | | | | | |
|---|---|---|---|---|---|---|---|---|
| Year＼Age | 20-29y | 30-39y | 40-49y | 50-59y | 60-69y | 70-79y | 80-89y | Total |
| 2000 | 21,542 | 42,518 | 77,665 | 136,622 | 184,241 | 194,445 | 103,742 | 760,775 |
| 2001 | 19,926 | 39,327 | 71,837 | 126,371 | 170,417 | 179,855 | 95,957 | 703,690 |
| 2002 | 18,431 | 36,376 | 66,447 | 116,888 | 157,629 | 166,360 | 88,757 | 650,888 |
| 2003 | 17,048 | 33,647 | 61,461 | 108,118 | 145,802 | 153,877 | 82,097 | 602,049 |
| 2004 | 15,769 | 31,122 | 56,849 | 100,005 | 134,861 | 142,331 | 75,937 | 556,873 |
| 2005 | 14,585 | 28,787 | 52,583 | 92,501 | 124,742 | 131,651 | 70,239 | 515,088 |
| 2006 | 13,491 | 26,627 | 48,638 | 85,560 | 115,382 | 121,772 | 64,969 | 476,438 |
| 2007 | 12,479 | 24,629 | 44,988 | 79,140 | 106,724 | 112,635 | 60,094 | 440,688 |
| 2008 | 11,542 | 22,781 | 41,613 | 73,202 | 98,716 | 104,183 | 55,585 | 407,621 |
| 2009 | 10,676 | 21,071 | 38,490 | 67,709 | 91,309 | 96,366 | 51,414 | 377,035 |
| 2010 | 9,875 | 19,490 | 35,602 | 62,628 | 84,457 | 89,135 | 47,556 | 348,744 |
| 2011 | 9,134 | 18,028 | 32,931 | 57,929 | 78,120 | 82,447 | 43,987 | 322,576 |
| 2012 | 8,449 | 16,675 | 30,460 | 53,582 | 72,258 | 76,260 | 40,687 | 298,371 |
| 2013 | 7,815 | 15,424 | 28,174 | 49,562 | 66,836 | 70,538 | 37,634 | 275,983 |
| 2014 | 7,228 | 14,267 | 26,060 | 45,843 | 61,821 | 65,245 | 34,810 | 255,274 |
| 2015 | 6,686 | 13,196 | 24,105 | 42,403 | 57,182 | 60,349 | 32,198 | 236,119 |
| 2016 | 6,184 | 12,206 | 22,296 | 39,221 | 52,892 | 55,821 | 29,782 | 218,402 |

| | | | | | | | | |
|---|---|---|---|---|---|---|---|---|
| **2017** | 5,720 | 11,290 | 20,623 | 36,278 | 48,923 | 51,633 | 27,547 | 202,014 |
| **2018** | 5,291 | 10,443 | 19,075 | 33,556 | 45,252 | 47,758 | 25,480 | 186,856 |
| **2019** | 4,894 | 9,659 | 17,644 | 31,038 | 41,856 | 44,175 | 23,568 | 172,835 |
| **2020** | 4,527 | 8,934 | 16,320 | 28,709 | 38,716 | 40,860 | 21,800 | 159,866 |
| **Total** | 231,292 | 456,496 | 833,861 | 1,466,865 | 1,978,137 | 2,087,696 | 1,113,841 | 8,168,187 |
| **Number of patients with duodenal ulcer (n)** | | | | | | | | |
| **Year＼Age** | **20-29y** | **30-39y** | **40-49y** | **50-59y** | **60-69y** | **70-79y** | **80-89y** | **Total** |
| **2000** | 3,658 | 11,583 | 23,167 | 28,654 | 27,435 | 23,167 | 11,583 | 129,247 |
| **2001** | 3,326 | 10,534 | 21,067 | 26,057 | 24,948 | 21,067 | 10,534 | 117,534 |
| **2002** | 3,025 | 9,579 | 19,158 | 23,696 | 22,687 | 19,158 | 9,579 | 106,882 |
| **2003** | 2,751 | 8,711 | 17,422 | 21,548 | 20,631 | 17,422 | 8,711 | 97,196 |
| **2004** | 2,502 | 7,921 | 15,843 | 19,595 | 18,761 | 15,843 | 7,921 | 88,387 |
| **2005** | 2,275 | 7,204 | 14,407 | 17,819 | 17,061 | 14,407 | 7,204 | 80,377 |
| **2006** | 2,069 | 6,551 | 13,102 | 16,204 | 15,515 | 13,102 | 6,551 | 73,092 |
| **2007** | 1,881 | 5,957 | 11,914 | 14,736 | 14,109 | 11,914 | 5,957 | 66,468 |
| **2008** | 1,711 | 5,417 | 10,834 | 13,400 | 12,830 | 10,834 | 5,417 | 60,445 |
| **2009** | 1,556 | 4,926 | 9,853 | 12,186 | 11,667 | 9,853 | 4,926 | 54,967 |
| **2010** | 1,415 | 4,480 | 8,960 | 11,082 | 10,610 | 8,960 | 4,480 | 49,985 |
| **2011** | 1,286 | 4,074 | 8,148 | 10,077 | 9,649 | 8,148 | 4,074 | 45,455 |
| **2012** | 1,170 | 3,705 | 7,409 | 9,164 | 8,774 | 7,409 | 3,705 | 41,336 |
| **2013** | 1,064 | 3,369 | 6,738 | 8,334 | 7,979 | 6,738 | 3,369 | 37,590 |
| **2014** | 967 | 3,064 | 6,127 | 7,578 | 7,256 | 6,127 | 3,064 | 34,183 |

| | | | | | | | | |
|---|---|---|---|---|---|---|---|---|
| **2015** | 880 | 2,786 | 5,572 | 6,891 | 6,598 | 5,572 | 2,786 | 31,085 |
| **2016** | 800 | 2,533 | 5,067 | 6,267 | 6,000 | 5,067 | 2,533 | 28,268 |
| **2017** | 728 | 2,304 | 4,608 | 5,699 | 5,456 | 4,608 | 2,304 | 25,706 |
| **2018** | 662 | 2,095 | 4,190 | 5,182 | 4,962 | 4,190 | 2,095 | 23,376 |
| **2019** | 602 | 1,905 | 3,810 | 4,713 | 4,512 | 3,810 | 1,905 | 21,258 |
| **2020** | 547 | 1,733 | 3,465 | 4,286 | 4,103 | 3,465 | 1,733 | 19,331 |
| **Total** | 34,873 | 110,430 | 220,860 | 273,169 | 261,545 | 220,860 | 110,430 | 1,232,167 |
